# Recent policy recommendations won’t protect young people from alcohol-related content on social media: what needs to change?

**DOI:** 10.1177/10398562251314692

**Published:** 2025-01-20

**Authors:** Maree Patsouras, Emmanuel Kuntsche, Amy Pennay, Paula O’Brien, Benjamin Riordan

**Affiliations:** Centre for Alcohol Policy Research, 2080 La Trobe University, Melbourne, VIC, Australia; Melbourne Law School, 2281 The University of Melbourne, Melbourne, VIC, Australia; Centre for Alcohol Policy Research, 2080 La Trobe University, Melbourne, VIC, Australia

**Keywords:** social media, alcohol use, policy, young people

## Abstract

**Objective:** The federal Australian government has introduced legislation to require social media platforms to restrict access to their platforms for young people under 16 years of age. Amongst the conversations about protecting the health and wellbeing of young people, we have yet to see discussion on the impact of alcohol imagery as a pervasive ‘unhealthy’ industry on social media. This is problematic because young people consume a large amount of social media content and are exposed to glamorised alcohol depictions and targeted advertising.

**Conclusions:** According to current regulations, the sponsoring of social media posts by alcohol companies should be declared, but enforcement of these requirements is challenging and most alcohol posts (whether sponsored or not) tend to glorify alcohol use. Better regulation, but not necessarily a social media ban, is needed to protect young viewers from pervasive alcohol exposure on social media.

Globally, there are concerns around the impact that social media can have on the health and wellbeing of young people. In response to these concerns, the Australian federal government has passed legislation requiring social media platforms to take reasonable steps to prevent persons under the age of 16 years from having accounts with those platforms.^1^ Social media platforms that are covered by the ‘social media ban’ but do not comply will run the risk of A$50 million in fines.^1^ Meta (the company that owns Instagram and Facebook) aimed to mitigate concerns before the legislation was passed, by introducing ‘Instagram Teen Accounts,’ with ‘built-in’ default limits on who can contact them, the content categories they can view, and parental supervision features.^2^ However, neither the ban nor Meta’s changes address the role and impact of exposure to alcohol-related content, which we believe should be a key aim for legislative change aiming to make these platforms safer for young people. In this viewpoint article, we review the evidence on the glamorisation of alcohol online and the impact of industry-sponsored influences, and we argue that alcohol-related content on social media warrants specific restrictions.

Among underage teenagers, the percentage of 14- to 17-year-olds consuming any alcohol in the past year has halved among both females (71% in 2001 vs 35% in 2022-2023) and males (67% in 2001 vs 27% in 2022-2023^3^). Although these declines are promising, it is important to note that alcohol remains a serious public health concern, responsible for 13% of annual deaths among Australians aged 14-17.^4,5^ This decline in young people’s drinking, and the risks associated with alcohol use, is not reflected on social media, where alcohol imagery is pervasive.

Globally, people spend an average of 143 minutes per day on social media.^6^ Young people are leading the way, with one American survey suggesting that teenagers aged 13-19 spend an average of 4.8 hours per day on social media platforms.^7^ As active consumers of digital and social media, young people are particularly susceptible to viewing alcohol-related content online.^4^ Alcohol is very common on social media,^8–10^ with an estimated 2% of Twitter posts referencing alcohol.^8^ Alcohol depictions online are also overwhelmingly positive and do not reflect the actual harms that alcohol can cause. For example, in an analysis of the top 100 TikTok #alcohol videos, 98% portrayed ‘pro-alcohol’ sentiment (e.g. enjoying alcohol), and 69% depicted positive alcohol experiences, with only 4% showing negative alcohol-related consequences.^9^ Positively oriented alcohol-related content also receives increased validation via engagement and likes, and can encourage other users to similarly post positive alcohol depictions online.^11–13^

In line with the Alcohol Self-Presentation Model, alcohol imagery may be predominantly positive because young people seek to cultivate online identities in either ‘protective’ (i.e. to preserve their image and avoid social disapproval) or ‘acquistive’ manners (i.e. to obtain validation, approval, and attention from others^11^). With the curated social media experience, user-generated posts continue to look positive because that is the nature of the platforms they are posting on.^11^

On top of being exposed to user-generated content, the World Health Organization highlights that young people are increasingly targeted by online alcohol marketing, at the expense of their health.^14^ The alcohol industry has embraced digital marketing, saturating the online environment with influencer sponsorship, targeted marketing, and product placement.^10,15^ Alcohol advertising is targeted towards young people with immediate short-term and insidious long-term objectives, aiming to increase sales, develop ‘drinking cultures’ resistant to change, and shape consumer behaviours and perceptions.^4,15^ Underage Australian minors are frequently exposed to social media alcohol advertising and targeted alcohol ads, and evidence has suggested that participants aged 17 (below the legal drinking age in Australia) were assigned alcohol-related interests via Facebook’s advertising model.^4,16^ The disconnect between alcohol’s prominent presence on social media and young people’s actual consumption rates may reflect the industry’s attempts to use its marketing to entice young people back to higher levels of drinking.

Participants in previous Australian research (including adult drinkers trying to reduce their consumption and parents) have expressed specific concerns about protecting the next ‘digital generation’ of children and teenagers from alcohol-related content online.^17^ This sample of Australian adults believed that viewing digital alcohol exposure was linked to increased temptation and craving of alcohol and an increased intent to purchase alcohol; and advocated for stricter governmental regulation of alcohol-related exposure and marketing.^17^ Alcohol marketing restrictions or bans by national governments have been recommended by the World Health Organization as one of the ‘best-buys’ to reduce alcohol harms (but strong enforcement is also needed^4^^;^^14^). The advantage that an alcohol marketing ban would have over Australia’s new ‘social media ban’ is that the problematic corporate marketing would be removed from social media completely, so that young people who access social media via their parents’ account or otherwise evade the ‘social media ban’ would not see alcohol marketing. This would still leave a large amount of user-generated alcohol content that is not marketing. However, another way to deal with such user-generated content would be to require media platforms to restrict access to certain alcohol-related content for those under age 18, such as through Sensitive Content Control as part of Meta’s new teen accounts or offer adult users the option to ‘opt in’ to reduce their alcohol exposure.^18^ Companies like Meta have highlighted that this is technically possible, by using technology to avoid recommending minors (under 18) content that promotes marijuana and non-medical drugs, even if they are following an account that posts this content.^18^ For example, content that depicts violence, tobacco, and vaping, or is sexually explicit or suggestive, is currently considered under ‘sensitive content’ by Instagram; it is not clear if this also includes alcohol, and we argue that it should.

It remains to be seen how effective or practical the social media ban will be for improving the health and wellbeing of Australians under the age of 16. By locking young people out of social media, we do not assist them to develop the digital literacy skills necessary to navigate these platforms in the future and in the context of alcohol, once someone turns 16, they are able to access an environment that is flooded with online alcohol content. One positive is that there appears to be an impetus from governments to introduce laws to reduce harms for young Australians; however, more specific alcohol policies and restrictions are required to protect young people from the excessive and glamorised portrayals of alcohol online.
